# Editorial: Creative metacognition: the chief manager of accurate decisions

**DOI:** 10.3389/fpsyg.2023.1210053

**Published:** 2023-05-10

**Authors:** Rogelio Puente-Díaz, Judith Cavazos-Arroyo, Alexander Brem

**Affiliations:** ^1^Department of Business and Economics, Universidad Anáhuac México, Huixquilucan, Estado de México, Mexico; ^2^Centro Interdisciplinario de Posgrados, Universidad Popular Autónoma del Estado de Puebla, Puebla, Mexico; ^3^Institute of Entrepreneurship and Innovation Science, University of Stuttgart, Stuttgart, Germany

**Keywords:** creative metacognition, creativity, innovation, accurate decision making, idea generation

Creativity is the key concept for our understanding of ideation and the consequent generation of innovations (Brem et al., 2016). Creative metacognition is the manager of cognitive functioning and accurate cognition in creativity (Fiedler et al., 2019). This is the proposition that drove our efforts to make a call for research on metacognition in creative problem-solving. Creative metacognition is probably one of the most dynamic, exciting themes of scientific inquiry in creativity research. For example, we have witnessed the development of a conceptual model of creative metacognition (Lebuda and Benedek, 2023), the examination of metacognitive feelings in idea evaluation and selection (Puente-Díaz, 2023), and the conceptualization of the dynamic nature of creativity (Beghetto and Karwowski, 2019), among others. In addition, evidence of the importance of accurate metacognition of the novelty and effectiveness of products and services continues to accumulate, as shown in some recent frauds (cryptocurrency company FTX and the sentencing of Elizabeth Holmes) coming from a place known for its outstanding creativity and innovation: Silicon Valley. There is a great deal of potential to increase our research efforts to understand creative metacognition and its implications for businesses, engineering, education, and society in general. Consequently, we posit three areas for future research based on the creativity literature in education and businesses before introducing six articles in this special issue.

## The role of emotions in creative metacognition

Thus far, research has focused on metacognitive feelings and their informative role in idea evaluation and selection (Puente-Díaz, 2023). The role of epistemic and achievement emotions has been neglected, even though these emotions are likely to play an important role in the creative process (Vogl et al., 2020). For example, emotions such as curiosity and interest are likely to influence the exploration process before individuals start generating ideas. The quality of the exploration process is likely to influence metacognitive judgments regarding how original and effective the ideas generated are (Agnoli and Corazza, 2019). Another epistemic emotion, such as satisfaction, would inform individuals about the need to continue generating and refining ideas or to stop. In addition, achievement emotions such as pride (Vogl et al., 2020) are also likely to influence idea evaluation and selection and possibly the socialization of ideas. Consequently, exploring epistemic and achievement emotions during the metacognitive processes involved in idea generation, evaluation, and selection represents an interesting research direction.

## The dynamic nature of creative metacognition

Individuals explore problems, generate ideas to solve them, evaluate, and select their ideas within a dynamic, constantly changing, evolving social environment. Even though studies using laboratory tasks have generated useful and relevant findings, we need more studies examining creative metacognition dynamically (Beghetto and Karwowski, 2019). As individuals start exploring the problem space, important metacognitive judgments are made regarding the relevance and depth of exploration. These judgments will likely influence the idea generation, evaluation, and selection processes. Similarly, the initial evaluation of ideas is likely to inform important decisions. Initial evaluations could come from the self, important others, and audiences in the form of potential consumers.

Acknowledging the dynamic of creative cognition would entail employing longitudinal designs (Beghetto and Karwowski, 2019), using mix-methods studies, combining qualitative and quantitative work (Creswell and Clark, 2018), and paying more attention to methodologies such as ethnography to examine creative cognition in the wild (Hutchins, 1995). Consequently, we make a call to increase the repertoire of methodologies used in creative metacognition to examine with more accuracy either a specific component or the whole creative process.

## The implications of accurate and inaccurate metacognition across domains

Inaccurate metacognition has direct consequences for individuals and societies. Different disciplines including education, business, innovation, public policy, entrepreneurship, and health could benefit from adopting a metacognitive approach to examine accurate and inaccurate decision-making. Accurate and inaccurate metacognition can be examined at two levels: intrapersonal and interpersonal. Regarding the intrapersonal level, individuals would benefit from having accurate metacognition. This personal epistemology (Ballantyne, 2019) would help individuals invest time in ideas that have potential novelty and effectiveness and disregard ideas with limited potential (Ballantyne and Dunning, 2022). More research is needed on the consequence of inaccurate and accurate metacognition at the intrapersonal level and in different domains such as business, education, engineering, and entrepreneurship.

The interpersonal level usually involves companies, organizations, and governments socializing the potential originality and effectiveness of the ideas in the form of new products, social policies, and investment opportunities. At this level, we could conceive genuine inaccurate creative metacognition and deception. Deceiving the public, financial investors and consumers could have legal ramifications in addition to the damage caused to laypeople. As of the writing of this article, there are more than four legal cases against several businesswomen and men for intentionally portraying their ideas as more original and effective than they really were (Griffith, 2023). While the act of committing fraud has many angles, we suggest that portraying ideas as more original and effective than they really are is often present, something known as business puffery. To our knowledge, the consequences of intentionally inaccurate communication of the potential originality and effectiveness of ideas have not received attention.

We are excited to briefly introduce readers to the six articles published in this special issue. The ability to shift between concepts to generate ideas, known as flexibility, could be conceptualized as a metacognitive monitoring process and decision. The article by Grajzel et al. introduces readers to a text-mining approach, showing the implications of machine learning for the automatic scoring of the flexibility of verbal responses in divergent thinking tasks. Addressing the importance of assessing creative metacognition in creative action, Von Thienen et al. introduce in their article a conceptual model of creative metacognition in design thinking, with important implications for future research. Similarly, Wang et al. represent in their article relevant developments in the assessment of awareness and self-evaluation with important implications for learning. In a fascinating cross-cultural study, Gai and Wang posit that meta-discourse is the linguistic representation of creative metacognition in writing. Creative metacognition helps individuals monitor and control their thinking process. This is extremely important when learners face uncertainty. Leclercq et al. introduce readers to the implications of metacognition for goal choice among preschoolers when facing unexpected changes. Last, Hong et al. address in their article the need to examine how emotions inform the creative process and show interesting results indicating that emotions interact with monitoring ability to predict creative problem-solving.

In sum, we are delighted to share six articles and this editorial with our fellow colleagues. We hope researchers find these contributions as insightful as we have as guest editors of this special issue.

## Author contributions

RP-D reviewed the literature and wrote the first and final draft. JC-A reviewed the literature and provided critical feedback. AB provided critical feedback. All authors contributed to the article and approved the submitted version.
